# Recurrent Cerebral Artery Dissection Associated with Seronegative Antiphospholipid Antibody Syndrome

**DOI:** 10.3390/tomography8020062

**Published:** 2022-03-10

**Authors:** Hee Sue Kim, Eun Su Lee, Byoung-Soo Shin, Hyun Goo Kang

**Affiliations:** 1 Jeonbuk National University Medical School, Jeonju 54907, Korea; helena3447@gmail.com; 2 Department of Neurology, Jeonbuk National University Medical School and Hospital, Jeonju 54907, Korea; rknnck0820@gmail.com (E.S.L.); sbsoo@jbnu.ac.kr (B.-S.S.); 3 Biomedical Research Institute, Jeonbuk National University Medical School and Hospital, Jeonju 54907, Korea

**Keywords:** arterial dissection, seronegative antiphospholipid syndrome, young age stroke

## Abstract

Stroke in young patients requires thorough evaluation as they often lack risk factors. Antiphospholipid syndrome can cause arterial thrombosis and pregnancy loss; hence, differential diagnoses should include seronegative antiphospholipid syndrome. We report a case of recurrent ischemic stroke caused by recurrent dissection in a patient with a history of pregnancy loss. A 33-year-old woman was admitted with global aphasia and right hemiparesis. During intra-arterial thrombectomy, a left middle cerebral artery dissection was detected. After 5.5 years, she was re-admitted for dysarthria, left facial palsy, subtle left hemiparesis, and right middle cerebral artery dissection. She tested negative for autoimmune diseases and vasculitis. However, underlying pathologic conditions could not be excluded because of the unique disease course. Finally, she was diagnosed with seronegative antiphospholipid syndrome. The concept of seronegative antiphospholipid syndrome has been proposed for patients with clinical features suggestive of antiphospholipid syndrome but with negative titers. However, this syndrome can only be diagnosed by exclusion. Furthermore, arterial dissection should be considered to be its main pathology. Antiphospholipid syndrome itself can be a risk factor for arterial dissection because it weakens the vessel walls. Therefore, diagnosis is important to prevent future complications in young patients with recurrent cerebral artery dissection, especially those associated with pregnancy-related morbidities.

## 1. Introduction

Stroke in young adults does not primarily occur due to atherosclerosis or cardioembolism, unlike most strokes occurring in older adults. Instead, the common causes of stroke in young adults include nonatherosclerotic angiopathies such as cervicocephalic arterial dissection and Moyamoya disease, and hematologic conditions such as hereditary thrombophilia and antiphospholipid syndrome (APS) [1]. Cervical arterial dissection is found in up to 25% of young patients with stroke [2]. However, it may be easily neglected during the initial diagnosis because it is quite infrequent, only accounting for 1% to 2% of all ischemic strokes.

Additionally, 20% of stroke cases in young adults are associated with APS [3]. Therefore, in cases with recurrent stroke, APS must be considered to be an underlying disease. Currently, if a patient displays a clinical course strongly suggestive of APS but shows negative titers based on the laboratory criteria, non-criteria antiphospholipid antibodies are considered and a diagnosis of seronegative APS (SN-APS) is reflected in treatment planning [4,5]. Previous studies also suggested a few non-criteria autoantibodies worth contemplating, such as antibodies against phosphatidylethanolamine (PE), phosphatidic acid (PA), phosphatidylserine (PS), phosphatidylinositol (PI), etc. [4] Thrombus formation from an abnormal coagulation cascade and complement activation is commonly known as the mechanism of stroke due to autoantibodies [3,6].

Moreover, there has been a case report on two different patients with stroke in which dissection and APS was observed simultaneously, suggesting a correlation between these conditions [7]. We encountered a case of recurrent intracranial arterial dissection at different locations in a patient with a history of miscarriages. She displayed a typical course of APS with negative laboratory test titers.

## 2. Case Presentation

**Figure 1:**
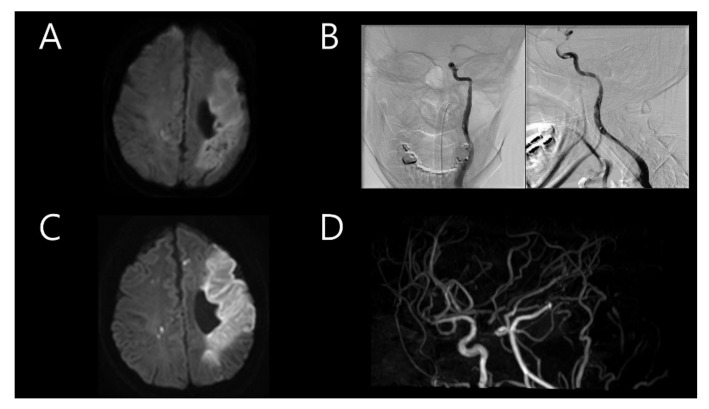
(**A**) Initial brain magnetic resonance imaging (MRI) after the first event showing stroke in the left middle cerebral artery (MCA) territory. (**B**) Immediate transfemoral cerebral angiography showing total occlusion of the distal portion of the left internal carotid artery (ICA). (**C**) Follow-up brain MRI on the following day showing acute infarction in the left MCA territory with multifocal embolic infarction in the bilateral hemispheres. (**D**) Magnetic resonance angiography showing complete occlusion of the left ICA.

A 33-year-old woman was previously admitted with global aphasia at gestational week 7. She received daily injections of 2 cc of progesterone and low-molecular-weight heparin due to her history of two previous pregnancy losses at 25 and 28 weeks of gestation, respectively. These miscarriages each happened 2 and 3 years before the stroke, spontaneously after cervical ripening. Neurological examination revealed alert mental status, global aphasia, and right hemiparesis (Medical Research Council (MRC) grade, 3/3), indicating a National Institute of Health Stroke Scale (NIHSS) score of 11. Brain magnetic resonance imaging (MRI) revealed a stroke in the left middle cerebral artery (MCA) territory (Figure 1A). Intravenous tissue plasminogen activator (tPA) was administered, with an onset-to-door time of 1 h and 23 min. Intra-arterial thrombectomy was also subsequently performed. Transfemoral cerebral angiography (TFCA) revealed the total occlusion of the distal portion of the left internal carotid artery (ICA) with a massive thrombus. It was clearly a by-product of arterial dissection nearby; significantly, the operator felt the existence of the flap even before any further invasive procedure was practiced. However, the procedure could not be continued because of the severe headache experienced by the patient (Figure 1B). A brain MRI performed the following day revealed acute infarction in the left MCA territory with multifocal embolic infarction in the bilateral hemispheres and the complete occlusion of the left ICA (Figure 1C,D). Transthoracic echocardiography (TTE) used to verify the cardioembolic source revealed mild akinesia; however, the heart appeared normal on cardiac computed tomography (CT). The cardiac CT findings suggested acute pulmonary thromboembolism in the left lower lobe. Considering the massive thrombus in the left MCA and suspected pulmonary thromboembolism, warfarin was administered. Autoimmune disease screening tests, including those for anticardiolipin antibody and anti-beta-2 glycoprotein I antibody, were conducted twice, with negative results both times. Additionally, coagulopathy screening tests, including those for antithrombin antibody 87.7% (normal range 75–125), protein C antigen 111.8% (normal range 72–160), and protein S antigen 108.4% (normal range 60–150), were negative. The patient was discharged after her neurological symptoms improved (NIHSS score of 3).

**Figure 2:**
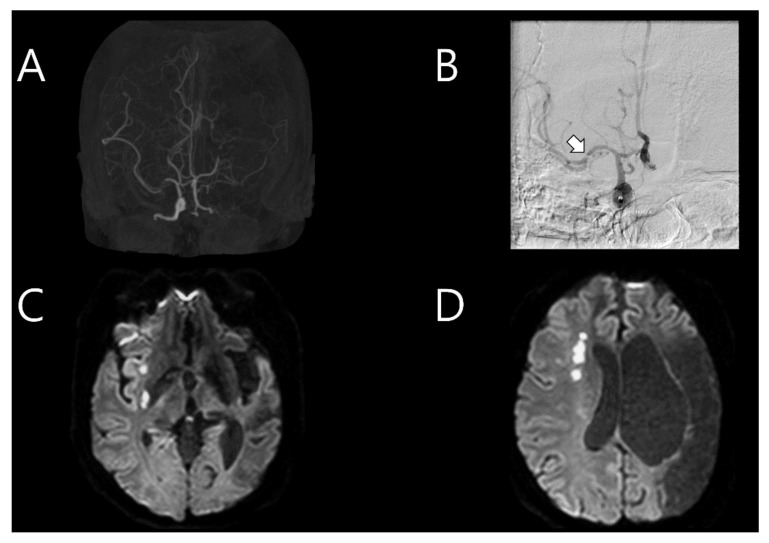
(**A**) Brain computed tomography angiography conducted on the second event which occurred after 5.5 years, showing recently formed severe stenosis in the M1 portion of the right middle cerebral artery (MCA), with left MCA occlusion present from the past stroke event. (**B**) Immediate cerebral angiography showing intramural hematoma and intimal flap in the M1 portion (arrow) of the right MCA. (**C**,**D**) Follow-up brain magnetic resonance imaging showing a multifocal acute infarction in the right MCA territory.

Five and a half years after the first stroke, the patient was re-admitted for sudden dysarthria. A neurological examination revealed alert mental status, mild dysarthria, left facial palsy, and left hemiparesis (MRC grade 4/4). Brain CT angiography indicated recent severe stenosis in the M1 portion of the right MCA, with previous occlusion in the left MCA (Figure 2A). Intravenous tPA was administered, with an onset-to-door time of 2 h and 20 min. Moreover, TFCA was conducted to examine the right MCA stenosis, which was distinct from the previous event, even though the patient did not display severe cortical symptoms. During right ICA angiography, intramural hematoma and intimal flap were detected in the M1 portion of the right MCA, indicating arterial dissection (Figure 2B). A brain MRI and CT performed after TFCA revealed multifocal acute infarction in the right MCA territory (Figure 2C,D). TTE did not reveal any significant changes compared to the previous examination. The results of screening tests for autoimmune diseases and vasculitis (antiphospholipid antibodies, lupus coagulant, and antinuclear antibody screening and all subtypes that could possibly be tested) to further evaluate the etiology of stroke in this young patient were all negative. The chromosome analysis result was also normal. Therefore, medication and rehabilitation therapies were administered.

## 3. Discussion

This case report describes a patient with a history of two spontaneous miscarriages who had been admitted for stroke. Dissection in the left distal ICA was detected during thrombectomy at that time. She had experienced no remarkable events for 5.5 years, when another stroke occurred due to right MCA dissection. Repetitive screening tests for autoimmune diseases and vasculitis were negative. The patient did not correspond to any of the reported risk factors of recurrent cervical arterial dissection; she had no family history of strokes, migraine, or hypertension; there was no evidence of connective tissue disorders or recent infections. Therefore, she was finally diagnosed with SN-APS based on her young age, recurrent dissection, and repeated pregnancy loss [4].

Stroke in young adults is less associated with the traditional risk factors of atherosclerosis and cardiovascular events. Therefore, “stroke of undetermined etiology” from the Trial of ORG 10172 in Acute Stroke Treatment (TOAST) classification is considered to be the most common cause [1]. Among the various causes, intra/extracranial artery dissection is one of the most representative underlying etiologies of stroke in young adults [2]. An intramural hematoma created in the false lumen travels through the arteries and may cause thromboembolism [8]. Connective tissue disease is one of the most classic non-traumatic risk factors of dissection that causes vessel wall weakness [8]. This case report suggests that an autoimmune disease, SN-APS, may have provoked recurrent cerebral arterial dissection by weakening the arterial walls.

**Figure 3:**
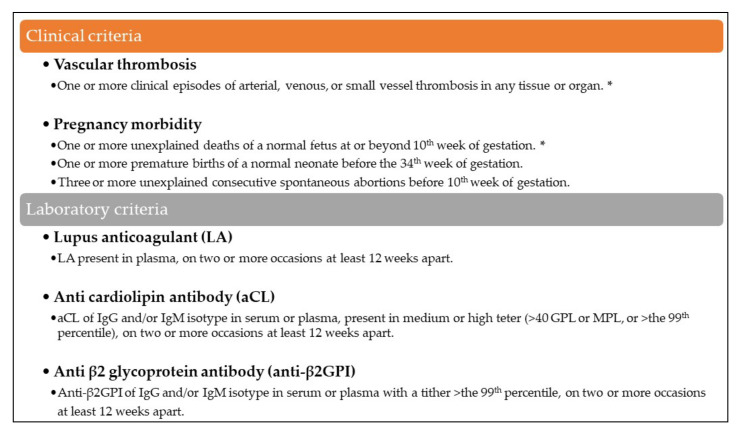
APS diagnosis criteria, updated at the Eleventh International Congress on Antiphospholipid Antibodies in Sydney in 2006. Antiphospholipid antibody syndrome (APS) is present if at least one of the clinical criteria and one of the laboratory criteria are met. Seronegative APS can be considered when a patient displays clinical features of APS with negative lab results. The clinical criteria that correspond the subject is marked (*).

APS is an autoimmune disease that presents thrombotic events and pregnancy-related morbidities with positive titers for anticardiolipin antibodies, anti-beta-2 glycoprotein-I antibodies, or lupus anticoagulant at the same time [9]. However, recent studies have described several patients with typical courses of APS, such as thrombotic events and repetitive pregnancy loss, despite negative results in antiphospholipid antibody screening tests [5,10]. Therefore, a new concept of SN-APS was proposed in 2003 that considered the presence of non-criteria antibodies against other phospholipids or protein co-factors [4,10]. A diagnosis of SN-APS may be considered in patients with a clinical course of classic APS in whom other probable causes of thrombophilia have been excluded [4]. Our patient was diagnosed with SN-APS due to her history of recurrent stroke and repetitive pregnancy loss (Figure 3), combined with the negative results of several screening tests for antiphospholipid antibodies and the exclusion of other coagulation disorders through blood coagulation tests. Moreover, the recurrence of dissection in different locations after a relatively long time strongly suggested an underlying autoimmune disease rather than spontaneous recurrence.

APS is one of the main risk factors of stroke in young adults, and the link between APS and dissection has been previously proposed [7,11]. However, earlier studies only revealed thrombus formation mechanism as a reason for stroke in APS; endothelial dysfunction due to autoantibodies appears as a “first hit”, and a subsequent inflammatory response as a “second hit” [12]. We propose that with a long-term perspective, accumulated endothelial dysfunction may also lead to dissection on fragile vessel walls. Autoantibodies downregulate endothelial nitric oxide synthase, resulting in a lack of nitric oxide formation. This reduces the flexibility of the vessel wall, increasing their vulnerability to damage [12]. The patient in the present case may have suffered recurrent dissection due to this vulnerability. Additionally, hypercoagulable state in APS patients may have a synergistic effect on large hematoma formation, thus increasing the risk of stroke when minimum dissection occurs. Considering these factors, we believe that non-criteria antibodies in the patient might have triggered the diverse mechanisms of APS and their complex interactions, leading to cerebral arterial dissection and massive thrombus formation.

As in this case, SN-APS should be considered to be a differential diagnosis in patients with a typical course of the disease, even though the detection method for non-criteria antibodies is not yet fully developed. This case report also suggests that cerebral arterial dissection should be considered to be the main cause of recurrent stroke in patients with APS. The mechanism is not simply thrombophilia causing thromboembolism; rather, APS itself can be a risk factor for arterial dissection. The limitation of this study remains since it cannot prove a time causal relationship between APS and arterial dissection. However, regarding the fact that untreated SN-APS resulted in separate events of dissection, the possibility should not be neglected. Therefore, SN-APS and dissection must be considered in young patients with a history of repetitive pregnancy loss exhibiting recurrent stroke and negative autoantibody screening test results.

## Data Availability

We can provide clinical data to editors upon appropriate request.
